# Public Mental Health System Resilience in Conflict Settings: Rebuilding Psychiatric Education and Nationwide Mental Health Care in Myanmar

**DOI:** 10.1192/bjo.2026.11611

**Published:** 2026-06-30

**Authors:** Maung Oakarr, Yan Lin Aung, Aung Zan, W Winnie, T Tina

**Affiliations:** Myanmar Board of Psychiatry, Rangoon, Myanmar

## Abstract

**Aims:**

**Background**

Armed conflict and political instability are major determinants of population mental health, disrupting service delivery, workforce sustainability, and prevention systems. Following the 2021 military coup in Myanmar, formal psychiatric training pathways and national mental health services were severely fragmented or collapsed. In response, the Myanmar Board of Psychiatry (M.B.Psych) was reconstituted in 2025 to support a population-oriented, task-shared mental health system operating under complex humanitarian conditions.

**Aims**

To describe a national public mental health model integrating workforce development, service delivery, prevention, and community-based interventions in a protracted conflict setting.

**Methods:**

A descriptive programme analysis was conducted using routine training, service delivery, and supervision data from M.B.Psych activities between January and December 2025. Interventions were mapped across core public mental health domains, including promotion, prevention, treatment, capacity building, and system governance.

**Results:**

M.B.Psych implemented a multi-layered mental health system spanning community, primary, and specialist care. Workforce capacity was strengthened through postgraduate psychiatric training, undergraduate teaching, medical education programmes, and international global mental health certification, with seven psychiatrists qualifying during the study period, supporting continuity of specialist care despite widespread displacement.

Population-level preventive and promotive interventions included nationwide Psychological First Aid, Mental Health and Psychosocial Support (MHPSS), mhGAP training, suicide prevention services, and school-based mental health programmes. Task-sharing enabled trained volunteers, lay counsellors, and primary care clinicians to deliver frontline care with structured psychiatric supervision.

Clinical services were delivered through an integrated hybrid model combining tele-mental health, primary mental health clinics, suicide prevention hotlines, and targeted on-ground psychiatric deployment in conflict-affected regions. Diaspora psychiatrists provided sustained supervision, psychotherapy training, and clinical governance support. Contextually adapted resources, including a Myanmar-language WHO Group PM+ manual and military-focused MHPSS materials, supported scalable and culturally appropriate interventions.

**Conclusion:**

This public mental health model demonstrates that equitable, population-based mental health systems can be sustained during armed conflict through task-sharing, digital delivery, diaspora engagement, and integrated education–service frameworks. The Myanmar experience offers transferable lessons for global mental health system strengthening in fragile and conflict-affected settings.

**Acknowledgements**

The authors acknowledge the commitment and resilience of all Civil Disobedience Movement (CDM) mental health professionals, psychiatrists, psychologists, trainees, volunteers, and the Myanmar psychiatrist diaspora who contributed to service delivery, training, supervision, and community mental health support under extremely challenging conditions.

